# Self-Assembling β-Glucan Nanomedicine for the Delivery of siRNA

**DOI:** 10.3390/biomedicines8110497

**Published:** 2020-11-12

**Authors:** Kyungwoo Lee, Daejin Min, Yonghyun Choi, Semi Yoon, Jaehee Jang, Jangsun Hwang, Hojeong Jeon, Yong Woo Cho, Jonghoon Choi

**Affiliations:** 1 School of Integrative Engineering, Chung-Ang University, Seoul 06974, Korea; orztapa@gmail.com (K.L.); dydgus5057@gmail.com (Y.C.); semi103306@gmail.com (S.Y.); jjaeh95@gmail.com (J.J.); 2 Center for Biomaterials, Korea Institute of Science and Technology, Seoul 02792, Korea; jeonhj@kist.re.kr; 3 BioScience Lab, Basic Research & Innovation Division, R&D Unit, AMOREPACIFIC, Yongin-si 17074, Korea; djmin@amorepacific.com; 4 Department of Chemical Engineering, Hanyang University, Ansan-si 15588, Korea; ywcho7@hanyang.ac.kr; 5 School of Chemical and Biomedical Engineering, Nanyang Technological University, Singapore 637457, Singapore; isnickawesome@gmail.com

**Keywords:** beta-glucan, self-assembly, nanomedicine, siRNA, gene delivery

## Abstract

We aimed to design and manufacture a transporter capable of delivering small interfering RNAs (siRNAs) into the skin without causing any damage. β-glucans are unique chiral polysaccharides with well-defined immunological properties and supramolecular wrapping ability. However, the chiral properties of these polymers have hardly been applied in drug delivery systems. In this study, β-glucan nanoparticles were designed and manufactured to deliver genetic material to the target cells. The β-glucan molecules were self-assembled with an siRNA into nanoparticles of 300–400 nm in diameter via a conformational transition process, in order to construct a gene delivery system. The assembled gene nanocarriers were associated with high gene-loading ability. The expression and efficiency of siRNA were verified after its delivery via β-glucan. Our results provide evidence that β-glucan nanoparticles can be effectively used to deliver siRNA into the cells.

## 1. Introduction

Small interfering RNAs (siRNAs) suppress the expression of the target gene and regulate it, thus suggesting a new possibility of their exploitation in gene therapy [1,2,3]. However, siRNAs have some disadvantages, such as a high molecular weight, difficulty in entering the cells, and easy degradation by enzymes. The development of siRNAs for practical application requires a technique for stably and effectively delivering the siRNAs to the target tissue. Research into siRNA delivery technology has mainly focused on the development of an input method and delivery system [4,5,6]. However, there are problems associated with siRNA delivery at various stages, depending on the route of delivery and the effect of the physical and biochemical environment of the disease [7,8,9].

The development of β-glucan nanocarriers has garnered increasing attention owing to its particular chiral interaction with bioactive molecules. The supramolecular interactions between β-glucans and pharmaceutical molecules are based on chiral interactions instead of excessive electrostatic/hydrophobic interactions, which are frequently adopted in conventional polymer drug delivery systems that may lead to denaturing of tissue proteins and damage to cell membranes [10,11,12,13,14]. Besides, β-glucan itself has a well-established immunopotentiation ability with low toxicity, as has been demonstrated in clinical trials [15,16,17,18]. The aggregation behavior of β-glucans in solution varies depending on their chain structures, which are also associated with their biological origin. Fungal β-glucan (schizophyllan) is relatively soluble in water and tends to exist in the form of well-dispersed triple helices. In previous research, self-assembled schizophyllan nanoparticles have been developed, and by treating these with trifluoroacetic acid, smaller size β-glucan nanoparticles (GluNPs) can be produced [12,14,17]. Helix-forming GluNPs and their derivatives have been extensively exploited to encapsulate drugs, as well as DNA and RNA molecules, and to deliver them to the target [15,19,20].

In a previous study, hollow β-glucan particles were prepared through a series of solvent extractions of β-glucan obtained from baker’s yeast. siRNA loading and an additional process of coating it with PEI (Polyethylenimine) polymer were required. The particles formed were 2–4 µm in size. In the present study, we employed a process of stepwise changes in pH during the β-glucan self-assembly that could produce nanosized carriers. Nanosized β-glucan carriers may help deliver genetic material more efficiently [21,22]. siRNA was loaded into the engineered GluNPs, after which the characteristics of the assembled nanomedicine were investigated. Its intracellular delivery capacity was evaluated by comparing it with a commercial gene carrier, Lipofectamine, and the degree of gene expression after delivery was also confirmed. We anticipate that this strategy, involving the delivery of genetic material using self-assembled GluNPs, which exhibit a low human toxicity and additional effects such as immune system activation, can be used in studies on siRNA delivery.

## 2. Results and Discussion

**Figure 1:**
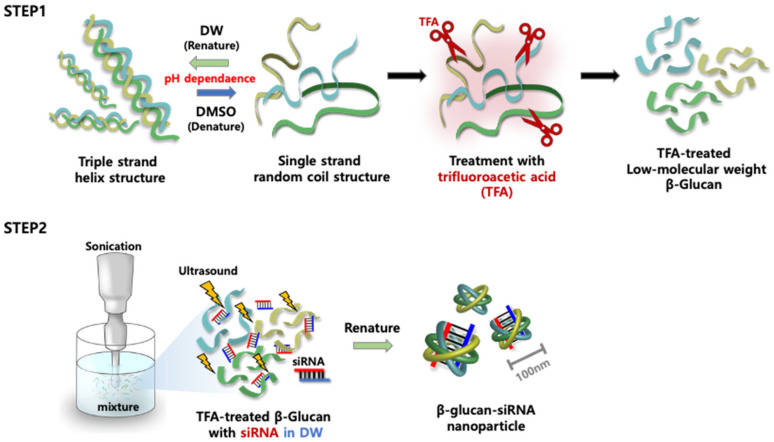
A schematic illustration of the preparation of siRNA-loaded β-glucan nanoparticles. Step 1 is preparing single strand β-glucan chains later cut with trifluoroacetic acid (TFA). Step 2 is promoting a binding reaction between siRNA and β-glucan structures. DW, deionized water; DMSO, dimethyl sulfoxide.

Figure 1 shows a schematic diagram of the overall method for constructing GluNPs, followed by siRNA loading. β-Glucan fibers dissolved in dimethyl sulfoxide (DMSO) create a single strand when their hydrophobic and hydrogen bonds are destroyed. In contrast, when mixed with water at a ratio of 1:20 or more, the dissolved single-stranded β-glucan structure becomes a triple helix (this process is called renaturation). At this point, the single-stranded fibers form granular particles while forming the natural triple-helix structure due to the formation of π, hydrogen, and hydrophobic bonds. In general, these β-granular glucan polymers, with a size of ~800 nm, can form smaller low-molecular-weight GluNPs by breaking the glycoside bonds with trifluoroacetic acid (TFA); the particle size varies according to the treatment ratio of TFA. In previous research, it was confirmed that a ratio of 1:4 was ideal, which was applied to subsequent experiments. siRNAs can be loaded into the low-molecular-weight GluNPs by forming hydrogen bonds between them.

**Figure 2:**
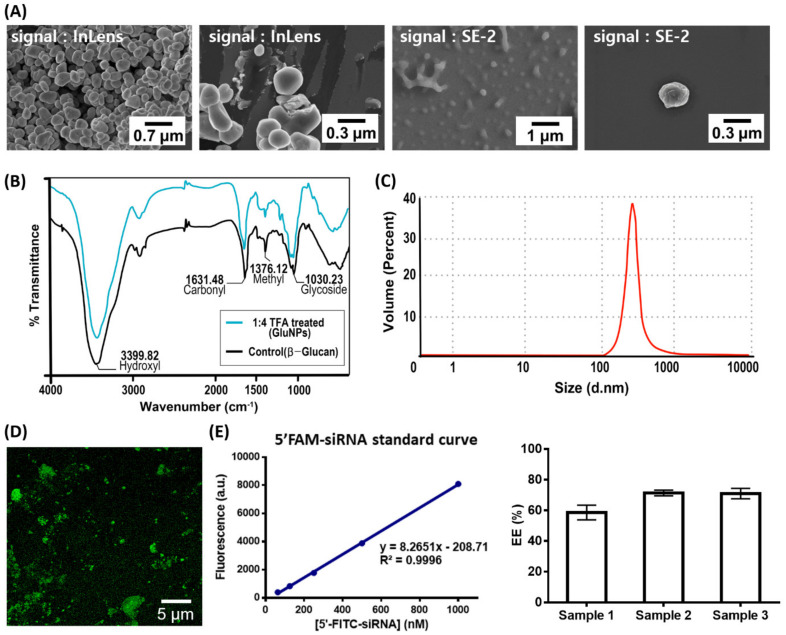
Characterization of the β-glucan nanoparticles (GluNPs) and siRNA entrapment efficiency (EE) evaluation. (**A**) Field emission-scanning electron microscopy analyses of the GluNPs using in-lens images taken at 1.6 kB; SE-2 images taken at 20 kV using a secondary electron microscope. (**B**) Fourier-transform infrared spectra of GluNPs (black) and TFA-treated GluNPs (red), and (**C**) size distribution of the GluNPs by volume measured using dynamic light scattering analysis. (**D**) Confocal microscopy analysis and (**E**) EE of three different samples (sample 1 (1:9 TFA treated), sample 2 (1:4 TFA treated), and sample 3 (1:1 TFA treated)) of 5′-FAM-siRNA/GluNPs calculated based on fluorescence measurements. The data are presented as mean ± SEM of three independent experiments. FAM, 5′-carboxyfluorescein; excitation max. = 495 nm, emission max. = 520 nm.

Figure 2 shows the results of scanning electron microscopy (SEM) and dynamic light scattering (DLS) analyses for the preparation of GluNPs as an siRNA carrier. The size of the GluNPs was 300 ± 50 nm. The organic properties of TFA can affect the functional groups on the surface of the GluNPs, which can act as a barrier to the synthesis and loading of the delivery material. Therefore, changes in the surface properties of GluNPs were analyzed using the Fourier-transform infrared (FT-IR) spectra (Figure 2B). The results showed that the peaks at 3399 cm^-1^, ~1631 cm^-1^, ~1376 cm^-1^, and ~1030 cm^-1^, corresponding to OH, C=O, CH, and CO (glycoside) stretching, respectively, were similar for the TFA-treated GluNPs (1:4 *v/v*) as well as the untreated control. These results confirm that there was no change in the surface properties of the GluNPs after treatment with TFA. siRNA entrapment in GluNPs was analyzed using confocal fluorescence microscopy on 5′-FAM-siRNA-loaded GluNPs (Figure 2D). The average EE of GluNP for siRNA was 67% for *n* = 3. The morphology of β-glucan can change from triple to a single strand depending on the pH, thereby allowing the desired material to be loaded through physical and/or chemical bonding. The target siRNA was successfully mounted on the nanocarrier through mutual hydrogen bonding and physical interaction between siRNA and β-glucan. Numerous studies have confirmed that β-glucan causes immune activity. In our previous study, the immune activity using β-glucan was confirmed. The FT-IR results in Figure 2B show that, even though acid treatment was performed, there was no change in the functional groups on the surface of β-glucan itself; therefore, the effect of the immunological activity of GluNP in this study was also considered to be significant.

Usually, β-glucan has a low solubility in aqueous solution; thus, aggregation is a reported problem [23,24]. In view of this, sonication and sufficient vortexing were performed. The confocal image in Figure 2D was obtained by inducing aggregation to some extent because of the properties of the nanoparticles. Homogeneously dispersed nanoparticles or excessive dilution treatment makes it difficult to obtain fluorescence signals of a single particle, and weak intensity signals are obtained as an image using a confocal microscope. Therefore, fluorescence-labeled siRNA in GluNP particles was loaded to analyze the fluorescence emitted from the aggregated particle mass.

In order to remove the residual siRNAs nonspecifically attached to the surface, the samples underwent sufficient washing via centrifugation three to four times after the loading of siRNA onto GluNP; the presence of nonspecifically attached siRNA on the surface was thus negligible.

**Figure 3:**
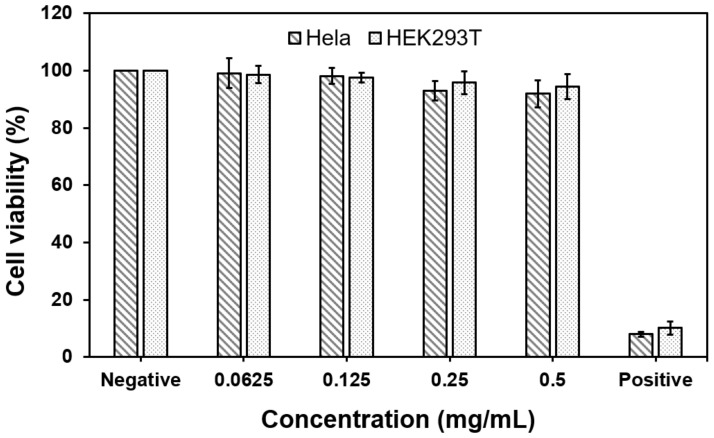
Cytotoxicity of varying concentrations of GluNPs toward HEK293T and HeLa cells. 50% DMSO was used as a positive control, and no treatment was used as negative control. The data are presented as mean ± SEM of three independent experiments. DMSO, dimethyl sulfoxide.

Figure 3 shows the results of toxicity evaluation of GluNPs in normal (HEK293T) and cancer (HeLa) cells. We used 50% DMSO as the positive control group, and no treatment was used as the negative control group. Both cell lines were cultured for 24 h with GluNPs to evaluate biocompatibility. Although the cells were almost killed by toxicity in the positive control group, the GluNPs were found to have little toxicity even at concentrations of up to 0.5 mg/mL, which was similar to the toxicity results of the negative control. The data are presented as mean ± SEM of three independent experiments.

**Figure 4:**
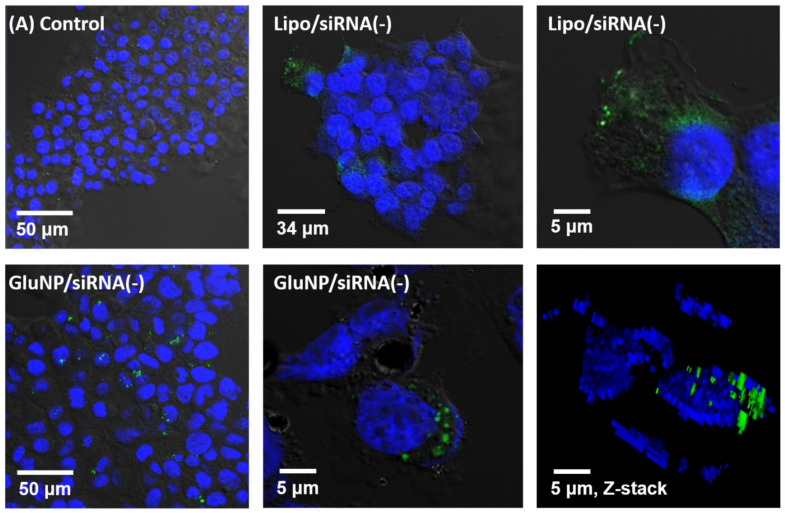
Cellular uptake of siRNA alone (negative control) or with Lipofectamine (positive control) or GluNPs (experimental group). Both Lipofectamine and GluNPs enabled the internalization of siRNA by the HEK293T cells.

Figure 4 shows the ability of GluNPs to enter the cells. For this experiment, GluNPs loaded with scrambled siRNA without the knockdown function were used as the negative control; the scrambled siRNA was labeled with 5′-FAM (excitation: 495 nm, emission: 520 nm). By treating the cells with GluNPs loaded with 5′-FAM-siRNA, we intended to observe its uptake by cells, through fluorescence analysis. The HEK293T cells are known to exhibit excellent transfection efficiency. The groups were prepared as follows: the negative control group was treated with free 5′-FAM-siRNA only, the positive control was treated with 5′-FAM-siRNA loaded using Lipofectamine RNAiMAX with high cell inflow and siRNA delivery efficiency, and the experimental group was prepared with 5′-FAM-siRNA/GluNPs. The results showed that there was no siRNA entry into the cell in the negative control group and that both the cytoplasm and the nucleus showed green fluorescence in the positive control. In the case of the experimental group, siRNA delivered into the cell was confirmed through green fluorescence. Although the experimental group was found to have an influx capacity into the cells, its efficiency was somewhat reduced because of particle aggregation. When the confocal Z-stack 3D image was analyzed, it was confirmed that GluNP has the ability to enter the cells (Figure 4). The intensity of fluorescence displayed does not indicate only the expression of a single particle. This is the result of aggregation of transporters containing a large amount of fluorescently expressed siRNA introduced into the cell. Note that it is not possible to directly count the number of single particles, including bubble-shaped liposomes such as Lipofectamine or nanoparticles such as GlunNP. Positive results for siRNA of the introduced carrier were confirmed using Western blotting.

GluNP (GluNP/siRNA) loaded with siRNA is recognized and bound by β-glucan receptors, such as Dectin-1 or TLR2, exposed on the surface of immune cells, such as macrophages and dendritic cells. Through this, endosomes are formed through fusion with the cell wall. siRNA from GluNP/siRNA in this complex must be released from the endosome into the intracellular cytosol. To analyze this escape mechanism of endosomes, many researchers use a strategy that induces the ‘proton sponge effect’ using a cation or a substance that induces a change in the pH in the carrier [25]. However, in this study, the GluNP/siRNA complex does not actively promote escape from the endosome. A previous study showed that numerous GluNP/siRNAs could be introduced into the cell, and GluNP/siRNA in the endosome occupies a significant portion. The endosome swells up because of the osmotic pressure caused by the depletion effect. In addition, as GluNP is fragmented at a low pH, siRNA may be separated and released into the cytoplasm of the cell [26].

HEK293T and HLEA cells were selected because they are highly efficient in transfection experiments and are commonly used in many studies. One of the goals of this study is to ensure that the complex could penetrate the skin; however, because it is important to obtain a therapeutic effect of siRNAs by delivering them to the cells, we experimentally selected cells with good efficiency to confirm this part.

**Figure 5:**
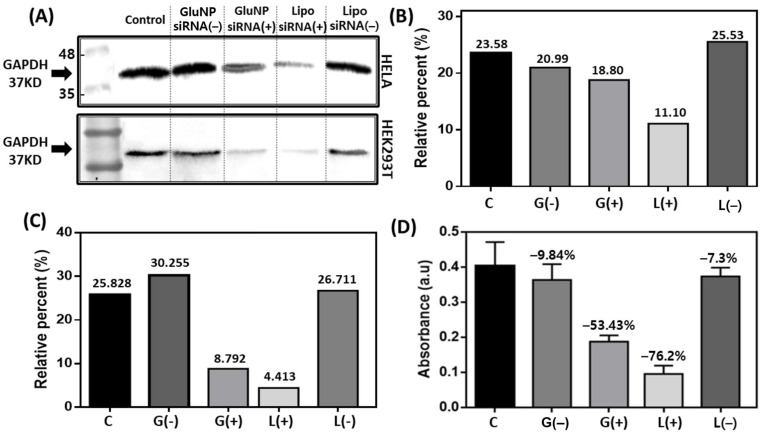
Delivery of siRNA using the β-glucan nanoparticles (GluNPs) to modulate the expression of glyceraldehyde 3-phosphate dehydrogenase (GAPDH). (**A**) Western blot results confirming the decreased expression of GAPDH in the HeLa and HEK293T cells treated with siRNA(−), siRNA/GluNP(−), siRNA/GluNP(+), Lipo (Lipofectamine)/siRNA(+), and Lipo/siRNA(−). Scrambled siRNA as the negative control (−). The positive control (+) siRNA could only knockdown the target gene GAPDH. Relative expression of GAPDH by (**B**) HeLa cells and (**C**) HEK293T cells plotted using Image J software to indicate the percentage of GAPDH expression area that is relative to the selected area including the five groups (double line of the box each cell). (**D**) Enzyme-linked immunosorbent assay (ELISA) of the lysed samples of HEK239T obtained from Western blot preparation confirmed the mRNA expression of GAPDH at the protein level. C, siRNA(−); G(−), siRNA/GluNP(−); G(+), siRNA/GluNP(+); L(+) Lipo/siRNA(+); L(−), Lipo/siRNA(−). The data are presented as mean ± SEM of three independent experiments.

Figure 5 shows the siRNA delivery mechanism and the knockdown efficiency after intracellular entry as observed using Western blotting and enzyme-linked immunosorbent assay (ELISA). To investigate the ability of siRNA knockdown using GlunNP, we initially chose the ubiquitously highly expressed housekeeping gene glyceraldehyde 3-phosphate dehydrogenase (GAPDH) as the target gene for knockdown. Transfection was carried out after loading siRNA into GluNPs and with Lipofectamine RNAiMAX. The negative control group was non-treated cells, whereas scrambled siRNA was used as the negative control for each carrier. In the Western blots, the intensity of the band according to gene expression was graphically expressed for each cell as the relative size of the band according to the total area (Figure 5B,C for HeLa and HEK293T, respectively). Although the knockdown effect of Lipofectamine seems to be significant in both types of cells, it can be seen that GluNP also exerted effects such as intracellular influx and siRNA delivery and knockdown at the intracellular level. In addition, ELISA of the GAPDH protein with the same residual samples showed similar results. At the cellular level, it was confirmed that both the GluNPs and Lipofectamine could be delivered intracellularly; the expression of GAPDH in the cells decreased, thereby displaying the knockdown effect, which is a function of siRNA. Lipofectamine is known to be a cytotoxic substance; thus, if the problem of aggregation of GluNP can be overcome, it is expected that the strategy presented here can be developed as an effective method for suppressing gene expression at the cellular level.

Generally, DNA has a negative charge because of its numerous phosphate groups. When DNA is introduced into the cells, the cell membrane, which has a negative charge, acts as a barrier. Cationic lipids bind to the negatively charged DNA and impart a net (+) charge; thus, DNA can be easily induced into the cell membrane. The DNA transfection reagent that uses this principle, lipofectamine, which was used as a control in this study, is a representative cationic lipid-based liposome, and has been used in many studies in gene delivery. On the other hand, in the case of GluNP, the loading of DNA (or siRNA) is not by charge interaction. DNA can be bound by the interaction between the pi bonds, hydrogen bonds, and hydrophobic bonds exposed during the exchange of single-triple helical structure of β-glucan [27].

## 3. Materials and Methods

Schizophyllan was purchased from Invitrogen (San Diego, CA, USA), and 5′-FAM-negative siRNA and GAPDH-positive siRNA were purchased from Bioneer (Seoul, Korea). Mouse GAPDH monoclonal antibodies, GAPDH-loading control monoclonal antibodies (the secondary antibody), and Lipofectamine™ RNAiMAX Transfection Reagent kit were purchased from Thermo Fisher Scientific (Waltham, MA, USA). Rabbit polyclonal anti-GAPDH primary antibody and anti-rabbit secondary anti-horseradish peroxidase (HRP) were purchased from Santa Cruz Biotechnology (Santa Cruz, CA, USA). Eight-well cell culture chamber slides were purchased from SPL life Sciences (Seoul, Korea). All other chemicals were purchased from Sigma-Aldrich (St. Louis, MO, USA) unless otherwise indicated.

### 3.1. Synthesis of GluNPs

First, 5 mg of schizophyllan polymer β-glucan powder was dissolved in 1 mL of dimethyl sulfoxide (DMSO) by stirring at 60 °C for 4 h. During this process, triple-helix β-glucan was converted into a single strand. Then, 100 μL of the β-glucan solution was taken; 900, 400, and 100 μL of trifluoroacetic acid (TFA 1:9, 1:4, 1:1 *v/v*) was added, and the mixture was stirred at 25 °C for 5 h. The reactants were transferred to a dialysis bag (a molecular weight cut-off of 4 kDa), immersed in excess deionized water, and then dialyzed for 3 days to remove the excess TFA. When it was confirmed that the dialyzed solution was near-neutral pH using pH paper, centrifugation was performed at 10,000× *g* for 30 min. Next, the supernatants were removed, and the pellets of GluNPs with a triple helical structure and low molecular weight were obtained.

### 3.2. Preparation of siRNA-Encapsulating GluNPs (siRNA/GluNPs)

The previously obtained pellets of GluNPs were dissolved in 1 mL of DMSO for 2 h at room temperature to convert the low-molecular-weight β-glucan into a single strand. After adding siRNA at a 10 nM final concentration, the mixture was stirred for 2 h. To produce nanosized GluNPs, 20 mL of deionized water (1:20 *v/v*) was added, followed by sonication for 1 h in an ultrasonic bath. Next, the reaction was continued in an orbital shaker for 12 h, and the final supernatant was removed by centrifugation at 10,000× *g* for 30 min to obtain siRNA/GluNPs; the samples were then stored at 4 °C until the next experiment. Meanwhile, the siRNA was labeled with 5′-carboxyfluorescein (FAM) at the 5′ end to visualize the siRNA/GluNPs.

### 3.3. Fluorescence Imaging and Quantification of 5′-FAM-siRNA

Confocal laser scanning microscopy (LSM710, Carl Zeiss, Oberkochen, Germany) was performed to visualize and quantify the 5′-FAM-siRNA in the siRNA/GluNPs. To confirm the entrapment efficiency (EE%) of siRNA in GluNPs, 5′-FAM-siRNA labeled with FAM fluorescent dye in GluNP was loaded. The concentration of free 5′-FAM-siRNA not bound to GluNP in the supernatant (supernatant) obtained by centrifugation in this process was calculated by plotting a standard curve of 5′-FAM-siRNA through fluorescence analysis. Using the following formula, the amount of siRNA loaded in GluNPs was quantified. The % EE for siRNA can be calculated as follows:

(1) $$EE\;\left(\%\right)=\frac{\left(C_{Final\;siRNA\;}-C_{Free\;supernatant}\right)}{C_{Final\;siRNA\;}}$$

where C = concentration (nM) and CFinal siRNA = 10 nM.

### 3.4. Field Emission-Scanning Electron Microscopy (FE-SEM)

Particle size and morphology were characterized using FE-SEM. Briefly, samples were mounted on carbon tape and ion-coated with Pt under vacuum using an ion coater current of 3 mA (COXEM, SPT-20). FE-SEM was performed on a JEM-F200 (JEOL, Tokyo, Japan) using an electron gun with an acceleration voltage of 5 kV.

### 3.5. Fourier-Transform Infrared (FTIR) Analysis

Schizophyllan (β-glucan), as the control, and TFA-treated GluNPs (β-glucan solution/TFA 1:4 *v/v*) were assessed via FT-IR (Jasco, Tokyo, Japan) to analyze the surface chemistry of each sample.

### 3.6. Dynamic Light Scattering (DLS)

Dynamic light scattering (Malvern, PA, USA) was also employed to analyze the size of β-glucan nanoparticles, for which samples were diluted to 1:100 in deionized water.

### 3.7. Cytotoxicity Testing

Cytotoxicity testing was conducted using the Cell counting kit (CCK)-8 assay (Dojindo, Tokyo, Japan), according to the manufacturer’s instructions. The normal cell line HEK293T and cancer cell line HeLa were seeded at 5 × 10^3^ cells per well onto a 96-well plate in Dulbecco’s modified Eagle’s medium (DMEM), containing 10% fetal bovine serum (Gibco life Science, Waltham, MA, USA) and 1% penicillin-streptomycin. The CCK-8 solution was used to determine the cell viability for 2 h with 5% CO_2_ at 37 °C after treatment with 0.0625, 0.125, 0.25, and 0.5 mg/mL of GluNPs at 37 °C under 5% CO_2_ over 24 h. The control group was not treated, and 50% DMSO was used as a positive control. After 2 h, 100 μL medium was transferred into a new 96-well plate, and the absorbance was measured at a wavelength of 450 nm using a Biomate 3S spectrophotometer (ThermoFisher, Waltham, MA, USA) to determine the maximum absorption wavelength of the GRs, PNIPAM/GR, and PNIPAM/GR-DOX.

### 3.8. Cellular Uptake of GluNPs

HEK293T cells (5 × 10^3^ cells/well) were seeded in an eight-well cell culture chamber slide and incubated overnight. The cells were exposed to GluNPs and Lipofectamine with 5′-FAM-siRNA at an siRNA dose of 10 nM, and incubated for 12 h. The control group was treated with free 5′-FAM-siRNA at the same concentration as the samples in Dulbecco’s phosphate-buffered saline (DPBS). Next, the cells were washed with DPBS and fixed with 4% paraformaldehyde for 1 min. The cells were stained with 4′,6-diamidino-2-phenylindole dihydrochloride (DAPI) to visualize the nucleus. Cell penetration of 5′-FAM-siRNA was then assessed via confocal laser scanning microscopy (LSM 710, Carl Zeiss, Oberkochen, Germany).

### 3.9. Cell Transfection and Gene Knockdown Experiments

HEK293T and HeLa cells (7.0 × 10^5^ cells/well) were seeded in six-well plates and incubated overnight at 37 °C under 5% CO_2_ before transfection. siRNA transfection using Lipofectamine RNAiMax (Invitrogen) was performed according to the forward siRNA transfection protocol, as per the manufacturer’s instructions (Thermo Fisher Scientific). The procedure was as follows: Lipofectamine plus scramble (control) siRNA, Lipofectamine plus GAPDH siRNA, GluNPs plus scramble (control) siRNA, GluNP plus GAPDH siRNA, and free GAPDH siRNA were employed as blank control. All culture media used in the following procedure were used with the reduced serum medium OPTI-MEM for diluting samples and effective transfection. For transfection of the positive controls, 15 μL of Lipofectamine RNAiMAX reagent and 80 pmol of siRNA were used to yield a final siRNA concentration of 50 nM. Furthermore, for transfection using GluNPs, the siRNA/GluNPs at the same concentration of siRNA were resuspended in the cell culture medium. After brief sonication of the siRNA/GluNPs, they were added to the cells along with Lipofectamine/siRNA. The medium was replaced after 6 h, and the transfection efficiency was evaluated after 24 h using Western blotting and enzyme-linked immunosorbent assay (ELISA).

### 3.10. Western Blotting

Whole cells were washed with PBS and lysed on a plate in ice-cold radioimmunoprecipitation assay (RIPA) buffer. The extracted pure protein was quantified using a Bio-Rad DC protein assay kit (Bio-Rad, Hercules, CA, USA) and a Synergy H1 spectrophotometer (BioTek Instruments, Winooski, VT, USA). Equal amounts (10 μg) of proteins were subjected to electrophoresis using 12% sodium dodecyl sulfate (SDS)-polyacrylamide gels followed by electrophoretic transfer of protein onto polyvinylidene difluoride (PVDF) membranes (Bio-Rad). To minimize any nonspecific binding of the primary or secondary antibodies to the membrane, the membrane had to be blocked. The blot was incubated in blocking buffer with gentle agitation at 4 °C overnight. The blocking buffer was removed, and the blot was washed thrice using Tris-buffered saline (TBS)-Tween for 5 min. The blot was incubated with rabbit polyclonal anti-GAPDH (diluted to 1:1000 in TBS-Tween) with gentle agitation for 2 h at room temperature, and then washed. After washing thrice, the blot was incubated with anti-rabbit secondary antibody linked to HRP with gentle agitation for 2 h at room temperature (diluted to 1:5000 in TBS-Tween). Protein band intensities were analyzed with a Chemidoc MP imager (Bio-Rad).

### 3.11. ELISA

After cell transfection, the indirect ELISA method was used to determine the expression of GAPDH with equal amounts of proteins obtained and quantified from lysed samples of HEK293T as those used for Western blotting. The primary antibody was monoclonal anti-GAPDH antibody from mice, and 2% bovine serum albumin (BSA) was used for blocking nonspecific binding. The secondary antibody was anti-mouse GAPDH-HRP used at a dilution ratio of 1:2000. Afterward, 2,20-azino-*bis*(3 -ethylbenzthiazoline-6-sulfonic acid) (ABTS) was used as a peroxidase substrate, and the absorbance at 410 nm was measured to determine the expression of GAPDH.

### 3.12. Statistical Analysis

Data are expressed as mean ± standard deviation (SD) of at least three independent determinations in triplicate for each experimental point.

## 4. Summary

siRNAs have considerable value in the treatment of diseases; however, it is challenging to deliver them to the target tissues, because of their large sizes. In this study, we produced biocompatible GluNPs capable of delivering siRNA to cells and confirmed the delivery and effects of siRNA in HeLa and HEK293T cells in vitro. In the future, we hope to use this skin-based delivery mechanism for gene therapy. It was shown that the size of the GluNPs could be reduced using TFA. We showed that the surface properties of β-glucan, when exposed to organic acids, were not lost. Moreover, GluNPs produced for siRNA delivery were shown to have excellent performance in gene delivery, with comparable efficacy to Lipofectamine. Finally, we evaluated whether the GluNPs loaded with siRNA could penetrate the surface of artificial skin tissues, but particles could not pass the skin layer, and this result is given in the Supplementary Material (Figure S1). Nucleic acid delivery through the skin via GluNPs requires improvement through further research. The skin barrier is virtually impermeable, and the way to penetrate the stratum corneum, which serves as the first defense line, should be considered first. To induce penetration through the skin via simple diffusion, it is necessary to produce even-sized smaller nanoparticles. In addition, skin permeation by electrostatic repulsion through the skin layers could be enhanced by controlling the surface modification of GluNPs.

## Supplementary Materials

The following are available online at https://www.mdpi.com/2227-9059/8/11/497/s1, Figure S1: The bright field and fluorescence microscopy images of organotypic 3D skin cryosection. Supporting information containing the artificial skin penetration mechanism of GluNPs is available.

## Abbreviations

5′-FAM	5-carboxyfluorescein
ABTS	2,20-azino-*bis*[3-ethylbenzthiazoline-6-sulfonic acid]
ANOVA	Analysis of variance
BSA	Bovine serum albumin
DAPI	4′6-diamidino-2-phenylindole dihydrochloride
DLS	Dynamic light scattering
DMSO	Dimethyl sulfoxide
DPBS	Dulbecco’s phosphate-buffered saline
EE	Entrapment efficiency
ELISA	Enzyme-linked immunosorbent assay
FE-SEM	Field emission-scanning electron microscopy
FTIR	Fourier-transform infrared
GAPDH	Glyceraldehyde 3-phosphate dehydrogenase
GluNP	β-glucan nanoparticle
HRP	Horseradish peroxidase
PBS	Phosphate-buffered saline
PVDF	Polyvinylidene difluoride
RIPA	Radioimmunoprecipitation assay
SDS	Sodium dodecyl sulfate
siRNA	Small interfering RNA
siRNA/GluNP	siRNA-encapsulated GluNP
TFA	Trifluoroacetic acid
