# Supplementary material for: Self-Assembling β-Glucan Nanomedicine for the Delivery of siRNA
Source: Biomedicines. 2020 Nov 12;8(11):497. doi: 10.3390/biomedicines8110497 (PMC7698166; doi:10.3390/biomedicines8110497)
Supplement: Supplementary file 1 [file biomedicines-08-00497-s001.pdf]

biomedicines

Supplementary Materials

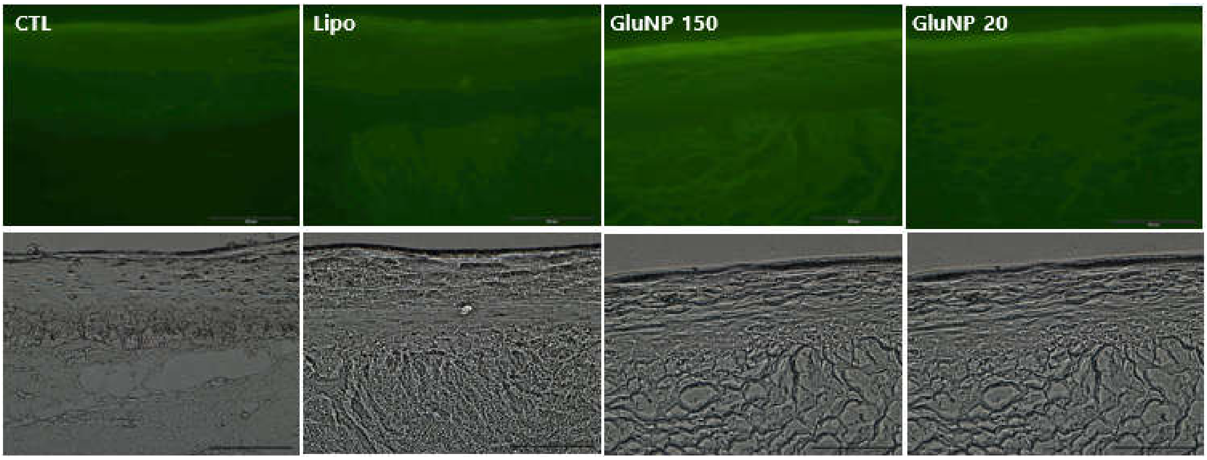

Figure S1. The bright field and fluorescence microscopy images of organotypic 3D skin cryosection with different volumes of GluNP/siRNA (20 and 150 μL) and Lipo/siRNA (20 μL). The control group (CTL) is treated with siRNA only. The siRNAs are labeled by 5′FAM (green).

Supplementary Method

Skin Penetration of GluNPs

Artificial skin tissue was provided by AMOREPACIFIC R&D Unit (Yongin-si, Republic of Korea). PBS (control, CTL), 5′-FAM-siRNA-loaded GluNPs (GluNP 20), and Lipofectamine/siRNA(Lipo) were loaded as an aliquot of 20 uL on the surface of the artificial skin tissue and incubated for 6 h at 37 °C in a humidified 5% CO_2 incubator. Additionally, we tried to add with more high volume of the GluNPs at 150 μL(GluNP 150). Afterwards, the surface of the artificial skin tissue was washed three times with PBS, and the artificial skin tissues were frozen for the cryosection. Then, 10 μm thick tissue sections were prepared and skin penetration was examined using fluorescent microscope (BX53, Olympus, Japan).
